# Chlorido[tris­(3-fluoro­phen­yl)phosphine]gold(I)

**DOI:** 10.1107/S1600536810034896

**Published:** 2010-09-08

**Authors:** Omar bin Shawkataly, Abu Tariq, Syed Sauban Ghani, Chin Sing Yeap, Hoong-Kun Fun

**Affiliations:** a Chemical Sciences Programme, School of Distance Education, Universiti Sains Malaysia, 11800 USM, Penang, Malaysia; b X-ray Crystallography Unit, School of Physics, Universiti Sains Malaysia, 11800 USM, Penang, Malaysia

## Abstract

In the title gold complex, [AuCl(C_18_H_12_F_3_P)], the P—Au—Cl unit is nearly linear, with an angle of 178.13 (5)°. The three phosphine-substituted benzene rings make dihedral angles of 77.7 (3), 84.4 (3) and 77.4 (3)° with each other. Two of the three F atoms are disordered over two positions, with refined site occupancies of 0.591 (11):0.409 (11) and 0.730 (12):0.270 (12). In the crystal structure, mol­ecules are linked into a three-dimensional network by inter­molecular C—H⋯Cl and C—H⋯F hydrogen bonds.

## Related literature

For general background to gold complex derivatives, see: Tiekink (2002 ▶); Dyadchenko (1982 ▶); Baenziger *et al.* (1976 ▶); Chen & Tiekink (2003 ▶). For the synthesis, see: Francis (1901 ▶). For the stability of the temperature controller used in the data collection, see: Cosier & Glazer (1986 ▶).
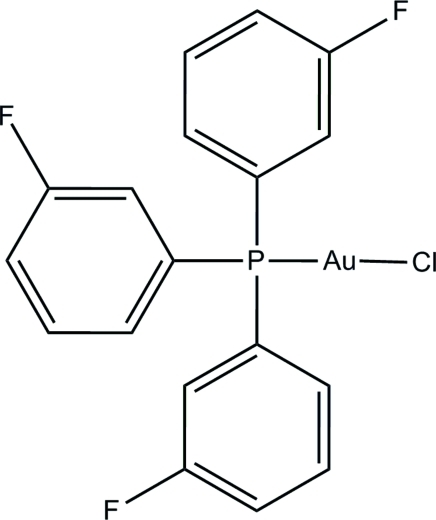


## Experimental

### 

#### Crystal data

[AuCl(C_18_H_12_F_3_P)]
*M* *_r_* = 548.66
Orthorhombic, 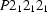

*a* = 10.4028 (8) Å
*b* = 12.3281 (11) Å
*c* = 13.2214 (10) Å
*V* = 1695.6 (2) Å^3^
*Z* = 4
Mo *K*α radiation
μ = 8.95 mm^−1^
*T* = 100 K
0.50 × 0.13 × 0.08 mm

#### Data collection

Bruker APEXII DUO CCD area-detector diffractometer
Absorption correction: multi-scan (*SADABS*; Bruker, 2009 ▶) *T*_min_ = 0.094, *T*_max_ = 0.520
14491 measured reflections
5912 independent reflections
5263 reflections with *I* > 2σ(*I*)
*R*_int_ = 0.031

#### Refinement

*R*[*F* ^2^ > 2σ(*F* ^2^)] = 0.025
*wR*(*F* ^2^) = 0.084
*S* = 1.06
5912 reflections
237 parameters
H-atom parameters constrained
Δρ_max_ = 1.25 e Å^−3^
Δρ_min_ = −0.87 e Å^−3^
Absolute structure: Flack (1983 ▶), 2522 Friedel pairs
Flack parameter: 0.010 (8)

### 

Data collection: *APEX2* (Bruker, 2009 ▶); cell refinement: *SAINT* (Bruker, 2009 ▶); data reduction: *SAINT*; program(s) used to solve structure: *SHELXTL* (Sheldrick, 2008 ▶); program(s) used to refine structure: *SHELXTL*; molecular graphics: *SHELXTL*; software used to prepare material for publication: *SHELXTL* and *PLATON* (Spek, 2009 ▶).

## Supplementary Material

Crystal structure: contains datablocks global, I. DOI: 10.1107/S1600536810034896/fj2329sup1.cif

Structure factors: contains datablocks I. DOI: 10.1107/S1600536810034896/fj2329Isup2.hkl

Additional supplementary materials:  crystallographic information; 3D view; checkCIF report

## Figures and Tables

**Table 1:** Hydrogen-bond geometry (Å, °)

*D*—H⋯*A*	*D*—H	H⋯*A*	*D*⋯*A*	*D*—H⋯*A*
C4—H4*A*⋯Cl1^i^	0.93	2.81	3.663 (6)	153
C5—H5*A*⋯Cl1^ii^	0.93	2.83	3.558 (7)	136
C10—H10*A*⋯F1^iii^	0.93	2.41	3.046 (8)	126

Symmetry codes: (i) 
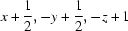
; (ii) 
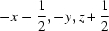
; (iii) 
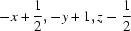
.

**Fig. 1.**
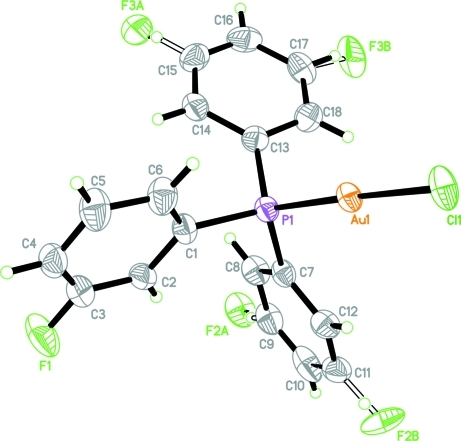
The molecular structure of the title compound with 50% probability ellipsoids for non-H atoms.

**Fig. 2.**
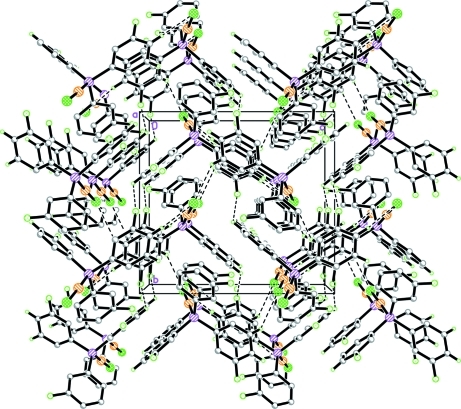
The crystal packing of the title compound, viewed down the a axis, showing the molecules linked into a 3-D network. Hydrogen atoms that not involved in the hydrogen-bonding (dashed lines) have been omitted for clarity.

## supplementary crystallographic information

### Comment

Gold complexes are well known for their medicinal (Tiekink, 2002) and catalytic activities (Dyadchenko, 1982). Phosphinegold(I) forms an important class of compounds of gold (Baenziger *et al.*, 1976). Chloro[tris(perflourophenyl)phospine]gold(I) is a known complex which is conveniently prepared and characterized (Chen & Tiekink, 2003). Keeping in mind the importance of the phosphine gold complexes, we have prepared the title complex and reported the crystal structure of the title complex.

In the title compound (Fig. 1), the P1–Au1–Cl1 is linear with an angle of 178.13 (5)°. The three phosphine substituted benzene rings (C1–C6, C7–C12 and C13–C18) make dihedral angles of 77.7 (3), 84.4 (3) and 77.4 (3)° with each other (C1–C6/C7–C12, C1–C6/C13–C18 and C7–C12/C13–C18). In the crystal structure, the molecules are linked into a three-dimensional network by intermolecular C4—H4A···Cl1, C5—H5A···Cl1 and C10—H10A···F1 hydrogen bonds (Fig. 2, Table 1).

### Experimental

The title compound was prepared from the reaction between Me_2_SAuCl (Francis, 1901) and (*m*-FC_6_H_4_)_3_P (Maybridge) in a 1:1 molar ratio in CH_2_Cl_2_ at room temperature. Solution was stirred for two hours, solvent was removed under vacuum, and white crystalline solid was obtained. The colorless crystals were obtained in 90% yield from the concentrated solution of the compound (m.p. 193 °C, decomposition) in ethanol kept for few days at room temperature.

### Refinement

All hydrogen atoms were positioned geometrically and refined using a riding model. Two out of the three flourine atoms were disordered over two positions with refined site occupancies of 0.591 (11)/409 (11) and 0.730 (12)/0.270 (12). The maximum and minimum residual electron density peaks of 1.25 and -0.87 e Å^-3^, respectively, were located 0.79 and 0.65 Å from the Au1 atom.

### Crystal data

**Table:**

[AuCl(C_18_H_12_F_3_P)]	*F*(000) = 1032
*M**_r_* = 548.66	*D*_x_ = 2.149 Mg m^−^^3^
Orthorhombic, *P*2_1_2_1_2_1_	Mo *K*α radiation, λ = 0.71073 Å
Hall symbol: P 2ac 2ab	Cell parameters from 7065 reflections
*a* = 10.4028 (8) Å	θ = 3.0–34.8°
*b* = 12.3281 (11) Å	µ = 8.95 mm^−^^1^
*c* = 13.2214 (10) Å	*T* = 100 K
*V* = 1695.6 (2) Å^3^	Block, colourless
*Z* = 4	0.50 × 0.13 × 0.08 mm

### Data collection

**Table:**

Bruker APEXII DUO CCD area-detector diffractometer	5912 independent reflections
Radiation source: fine-focus sealed tube	5263 reflections with *I* > 2σ(*I*)
graphite	*R*_int_ = 0.031
φ and ω scans	θ_max_ = 32.5°, θ_min_ = 2.3°
Absorption correction: multi-scan (*SADABS*; Bruker, 2009)	*h* = −15→15
*T*_min_ = 0.094, *T*_max_ = 0.520	*k* = −18→18
14491 measured reflections	*l* = −19→19

### Refinement

**Table:**

Refinement on *F*^2^	Secondary atom site location: difference Fourier map
Least-squares matrix: full	Hydrogen site location: inferred from neighbouring sites
*R*[*F*^2^ > 2σ(*F*^2^)] = 0.025	H-atom parameters constrained
*wR*(*F*^2^) = 0.084	*w* = 1/[σ^2^(*F*_o_^2^) + (0.0362*P*)^2^] where *P* = (*F*_o_^2^ + 2*F*_c_^2^)/3
*S* = 1.06	(Δ/σ)_max_ < 0.001
5912 reflections	Δρ_max_ = 1.25 e Å^−^^3^
237 parameters	Δρ_min_ = −0.87 e Å^−^^3^
0 restraints	Absolute structure: Flack (1983), 2522 Friedel pairs
Primary atom site location: structure-invariant direct methods	Flack parameter: 0.010 (8)

### Special details

**Table:**

Experimental. The crystal was placed in the cold stream of an Oxford Cryosystems Cobra open-flow nitrogen cryostat (Cosier & Glazer, 1986) operating at 100.0 (1) K.
Geometry. All e.s.d.'s (except the e.s.d. in the dihedral angle between two l.s. planes) are estimated using the full covariance matrix. The cell e.s.d.'s are taken into account individually in the estimation of e.s.d.'s in distances, angles and torsion angles; correlations between e.s.d.'s in cell parameters are only used when they are defined by crystal symmetry. An approximate (isotropic) treatment of cell e.s.d.'s is used for estimating e.s.d.'s involving l.s. planes.
Refinement. Refinement of *F*^2^ against ALL reflections. The weighted *R*-factor *wR* and goodness of fit *S* are based on *F*^2^, conventional *R*-factors *R* are based on *F*, with *F* set to zero for negative *F*^2^. The threshold expression of *F*^2^ > σ(*F*^2^) is used only for calculating *R*-factors(gt) *etc*. and is not relevant to the choice of reflections for refinement. *R*-factors based on *F*^2^ are statistically about twice as large as those based on *F*, and *R*- factors based on ALL data will be even larger.

### Fractional atomic coordinates and isotropic or equivalent isotropic displacement parameters (Å^2^)

**Table:**

	*x*	*y*	*z*	*U*_iso_*/*U*_eq_	Occ. (<1)
Au1	−0.172736 (12)	0.064703 (12)	0.248330 (12)	0.02814 (5)	
Cl1	−0.37503 (10)	0.00469 (13)	0.21314 (9)	0.0430 (3)	
P1	0.02323 (11)	0.12844 (9)	0.28033 (8)	0.02683 (19)	
F1	0.1479 (5)	0.4535 (3)	0.5012 (3)	0.0647 (11)	
F2A	0.3891 (5)	0.3156 (6)	0.0912 (4)	0.0506 (19)	0.591 (11)
F3A	0.3834 (5)	−0.0758 (4)	0.4689 (4)	0.0577 (17)	0.730 (12)
F2B	−0.0532 (9)	0.3838 (8)	−0.0232 (7)	0.057 (3)	0.409 (11)
F3B	0.2739 (12)	−0.1835 (13)	0.1292 (11)	0.058 (5)	0.270 (12)
C1	0.0268 (5)	0.2072 (4)	0.3955 (3)	0.0314 (8)	
C2	0.0940 (5)	0.3051 (4)	0.4037 (4)	0.0339 (9)	
H2A	0.1430	0.3313	0.3502	0.041*	
C3	0.0859 (6)	0.3613 (5)	0.4928 (4)	0.0415 (11)	
C4	0.0171 (5)	0.3264 (5)	0.5753 (4)	0.0403 (11)	
H4A	0.0139	0.3670	0.6345	0.048*	
C5	−0.0473 (7)	0.2285 (6)	0.5669 (4)	0.0503 (14)	
H5A	−0.0916	0.2009	0.6222	0.060*	
C6	−0.0460 (5)	0.1720 (6)	0.4773 (4)	0.0423 (12)	
H6A	−0.0948	0.1091	0.4713	0.051*	
C7	0.0838 (4)	0.2165 (4)	0.1821 (3)	0.0292 (8)	
C8	0.2166 (5)	0.2331 (4)	0.1705 (4)	0.0369 (10)	
H8A	0.2759	0.1980	0.2118	0.044*	
C9	0.2563 (5)	0.3051 (5)	0.0936 (4)	0.0412 (11)	
H9A	0.3436	0.3195	0.0863	0.049*	0.409 (11)
C10	0.1742 (7)	0.3530 (5)	0.0314 (4)	0.0493 (15)	
H10A	0.2039	0.3980	−0.0199	0.059*	
C11	0.0419 (6)	0.3350 (4)	0.0439 (4)	0.0398 (11)	
H11A	−0.0164	0.3693	0.0012	0.048*	0.591 (11)
C12	−0.0014 (4)	0.2683 (4)	0.1172 (4)	0.0362 (10)	
H12A	−0.0893	0.2569	0.1244	0.043*	
C13	0.1464 (4)	0.0259 (4)	0.2926 (3)	0.0308 (8)	
C14	0.2201 (5)	0.0134 (4)	0.3816 (4)	0.0360 (10)	
H14A	0.2075	0.0577	0.4377	0.043*	
C15	0.3122 (5)	−0.0682 (5)	0.3812 (4)	0.0434 (12)	
H15A	0.3621	−0.0769	0.4390	0.052*	0.270 (12)
C16	0.3342 (5)	−0.1358 (5)	0.3023 (5)	0.0448 (12)	
H16A	0.3983	−0.1883	0.3057	0.054*	
C17	0.2581 (5)	−0.1250 (5)	0.2156 (5)	0.0428 (11)	
H17A	0.2706	−0.1714	0.1610	0.051*	0.730 (12)
C18	0.1647 (5)	−0.0455 (5)	0.2107 (4)	0.0377 (10)	
H18A	0.1138	−0.0392	0.1532	0.045*	

### Atomic displacement parameters (Å^2^)

**Table:**

	*U*^11^	*U*^22^	*U*^33^	*U*^12^	*U*^13^	*U*^23^
Au1	0.02793 (7)	0.02848 (8)	0.02799 (7)	−0.00558 (5)	0.00235 (8)	−0.00385 (9)
Cl1	0.0336 (5)	0.0533 (7)	0.0421 (5)	−0.0160 (5)	0.0050 (4)	−0.0161 (6)
P1	0.0275 (4)	0.0269 (5)	0.0262 (4)	−0.0036 (4)	0.0003 (4)	−0.0018 (4)
F1	0.091 (3)	0.047 (2)	0.0557 (19)	−0.034 (2)	0.014 (2)	−0.0163 (18)
F2A	0.048 (3)	0.053 (4)	0.051 (3)	−0.020 (3)	0.014 (2)	−0.004 (3)
F3A	0.067 (4)	0.045 (3)	0.061 (3)	0.003 (2)	−0.026 (3)	0.004 (2)
F2B	0.062 (6)	0.058 (6)	0.050 (5)	0.017 (5)	0.006 (4)	0.036 (4)
F3B	0.050 (7)	0.061 (9)	0.064 (8)	0.020 (6)	−0.001 (6)	−0.043 (8)
C1	0.0306 (17)	0.034 (2)	0.0297 (18)	0.0007 (19)	−0.0011 (16)	−0.0077 (17)
C2	0.044 (2)	0.027 (2)	0.0308 (19)	−0.004 (2)	0.0000 (17)	0.0008 (19)
C3	0.049 (3)	0.035 (3)	0.040 (2)	−0.006 (2)	−0.006 (2)	−0.005 (2)
C4	0.045 (2)	0.042 (3)	0.034 (2)	0.006 (2)	−0.0003 (19)	−0.010 (2)
C5	0.055 (3)	0.062 (4)	0.034 (2)	−0.005 (3)	0.006 (2)	−0.010 (3)
C6	0.042 (3)	0.052 (3)	0.032 (2)	−0.011 (2)	0.0030 (19)	−0.002 (2)
C7	0.0319 (18)	0.030 (2)	0.0260 (17)	−0.0060 (16)	0.0048 (14)	−0.0041 (16)
C8	0.034 (2)	0.038 (2)	0.039 (2)	−0.009 (2)	0.0085 (18)	−0.002 (2)
C9	0.046 (3)	0.042 (3)	0.036 (2)	−0.014 (2)	0.0097 (19)	−0.005 (2)
C10	0.067 (4)	0.044 (3)	0.036 (2)	−0.025 (3)	0.020 (2)	−0.012 (2)
C11	0.057 (3)	0.030 (2)	0.033 (2)	−0.001 (2)	0.003 (2)	0.0019 (19)
C12	0.039 (3)	0.033 (2)	0.037 (2)	−0.0033 (19)	0.0061 (17)	−0.002 (2)
C13	0.0290 (17)	0.030 (2)	0.0330 (19)	−0.0024 (16)	0.0018 (15)	0.0014 (17)
C14	0.037 (2)	0.032 (2)	0.039 (2)	−0.0041 (19)	−0.0041 (18)	−0.003 (2)
C15	0.037 (2)	0.045 (3)	0.048 (3)	−0.005 (2)	−0.011 (2)	0.011 (2)
C16	0.034 (2)	0.036 (3)	0.065 (3)	0.005 (2)	0.001 (2)	0.009 (3)
C17	0.040 (2)	0.039 (3)	0.050 (3)	−0.002 (2)	0.005 (2)	−0.013 (2)
C18	0.036 (2)	0.043 (3)	0.035 (2)	0.001 (2)	0.0001 (17)	0.001 (2)

### Geometric parameters (Å, °)

**Table:**

Au1—P1	2.2254 (11)	C7—C8	1.405 (6)
Au1—Cl1	2.2787 (11)	C8—C9	1.412 (7)
P1—C1	1.806 (4)	C8—H8A	0.9300
P1—C7	1.807 (5)	C9—C10	1.324 (9)
P1—C13	1.807 (5)	C9—H9A	0.9300
F1—C3	1.312 (7)	C10—C11	1.404 (9)
F2A—C9	1.387 (8)	C10—H10A	0.9300
F3A—C15	1.379 (7)	C11—C12	1.348 (7)
F2B—C11	1.458 (10)	C11—H11A	0.9300
F3B—C17	1.360 (12)	C12—H12A	0.9300
C1—C6	1.390 (7)	C13—C18	1.409 (7)
C1—C2	1.399 (7)	C13—C14	1.413 (7)
C2—C3	1.369 (7)	C14—C15	1.389 (8)
C2—H2A	0.9300	C14—H14A	0.9300
C3—C4	1.373 (8)	C15—C16	1.355 (9)
C4—C5	1.385 (9)	C15—H15A	0.9300
C4—H4A	0.9300	C16—C17	1.399 (9)
C5—C6	1.375 (8)	C16—H16A	0.9300
C5—H5A	0.9300	C17—C18	1.381 (8)
C6—H6A	0.9300	C17—H17A	0.9300
C7—C12	1.390 (7)	C18—H18A	0.9300
			
P1—Au1—Cl1	178.13 (5)	C8—C9—H9A	118.7
C1—P1—C7	106.0 (2)	C9—C10—C11	119.3 (5)
C1—P1—C13	106.6 (2)	C9—C10—H10A	120.3
C7—P1—C13	103.7 (2)	C11—C10—H10A	120.3
C1—P1—Au1	111.66 (16)	C12—C11—C10	120.5 (6)
C7—P1—Au1	113.28 (15)	C12—C11—F2B	117.5 (6)
C13—P1—Au1	114.81 (15)	C10—C11—F2B	121.9 (6)
C6—C1—C2	118.8 (5)	C12—C11—H11A	119.7
C6—C1—P1	118.4 (4)	C10—C11—H11A	119.7
C2—C1—P1	122.7 (4)	C11—C12—C7	120.7 (5)
C3—C2—C1	118.2 (5)	C11—C12—H12A	119.6
C3—C2—H2A	120.9	C7—C12—H12A	119.6
C1—C2—H2A	120.9	C18—C13—C14	119.9 (4)
F1—C3—C2	118.7 (5)	C18—C13—P1	117.7 (3)
F1—C3—C4	117.4 (5)	C14—C13—P1	122.4 (4)
C2—C3—C4	123.9 (5)	C15—C14—C13	116.8 (5)
C3—C4—C5	117.4 (5)	C15—C14—H14A	121.6
C3—C4—H4A	121.3	C13—C14—H14A	121.6
C5—C4—H4A	121.3	C16—C15—F3A	121.0 (5)
C6—C5—C4	120.5 (6)	C16—C15—C14	124.4 (5)
C6—C5—H5A	119.8	F3A—C15—C14	114.7 (6)
C4—C5—H5A	119.8	C16—C15—H15A	117.8
C5—C6—C1	121.1 (6)	C14—C15—H15A	117.8
C5—C6—H6A	119.4	C15—C16—C17	118.5 (5)
C1—C6—H6A	119.4	C15—C16—H16A	120.8
C12—C7—C8	119.6 (4)	C17—C16—H16A	120.8
C12—C7—P1	119.8 (3)	F3B—C17—C18	114.9 (8)
C8—C7—P1	120.6 (4)	F3B—C17—C16	124.7 (8)
C7—C8—C9	117.2 (5)	C18—C17—C16	120.3 (5)
C7—C8—H8A	121.4	C18—C17—H17A	119.9
C9—C8—H8A	121.4	C16—C17—H17A	119.9
C10—C9—F2A	125.9 (6)	C17—C18—C13	120.1 (5)
C10—C9—C8	122.6 (5)	C17—C18—H18A	119.9
F2A—C9—C8	111.5 (6)	C13—C18—H18A	119.9
C10—C9—H9A	118.7		
			
C7—P1—C1—C6	162.5 (4)	F2A—C9—C10—C11	−179.5 (6)
C13—P1—C1—C6	−87.4 (5)	C8—C9—C10—C11	2.2 (9)
Au1—P1—C1—C6	38.7 (5)	C9—C10—C11—C12	−1.1 (9)
C7—P1—C1—C2	−13.8 (5)	C9—C10—C11—F2B	−178.9 (7)
C13—P1—C1—C2	96.2 (4)	C10—C11—C12—C7	0.3 (8)
Au1—P1—C1—C2	−137.6 (4)	F2B—C11—C12—C7	178.2 (6)
C6—C1—C2—C3	0.7 (8)	C8—C7—C12—C11	−0.6 (8)
P1—C1—C2—C3	177.0 (4)	P1—C7—C12—C11	179.5 (4)
C1—C2—C3—F1	−179.6 (5)	C1—P1—C13—C18	−178.8 (4)
C1—C2—C3—C4	1.0 (9)	C7—P1—C13—C18	−67.1 (4)
F1—C3—C4—C5	−179.3 (6)	Au1—P1—C13—C18	57.0 (4)
C2—C3—C4—C5	0.0 (9)	C1—P1—C13—C14	3.4 (5)
C3—C4—C5—C6	−2.9 (10)	C7—P1—C13—C14	115.1 (4)
C4—C5—C6—C1	4.6 (10)	Au1—P1—C13—C14	−120.8 (4)
C2—C1—C6—C5	−3.5 (9)	C18—C13—C14—C15	2.6 (7)
P1—C1—C6—C5	−180.0 (5)	P1—C13—C14—C15	−179.7 (4)
C1—P1—C7—C12	−100.3 (4)	C13—C14—C15—C16	−0.6 (8)
C13—P1—C7—C12	147.6 (4)	C13—C14—C15—F3A	178.7 (5)
Au1—P1—C7—C12	22.4 (4)	F3A—C15—C16—C17	179.5 (5)
C1—P1—C7—C8	79.8 (4)	C14—C15—C16—C17	−1.2 (8)
C13—P1—C7—C8	−32.3 (5)	C15—C16—C17—F3B	176.5 (10)
Au1—P1—C7—C8	−157.4 (4)	C15—C16—C17—C18	1.2 (8)
C12—C7—C8—C9	1.6 (7)	F3B—C17—C18—C13	−175.0 (9)
P1—C7—C8—C9	−178.5 (4)	C16—C17—C18—C13	0.8 (8)
C7—C8—C9—C10	−2.5 (8)	C14—C13—C18—C17	−2.7 (7)
C7—C8—C9—F2A	179.0 (5)	P1—C13—C18—C17	179.5 (4)

### Hydrogen-bond geometry (Å, °)

**Table:**

*D*—H···*A*	*D*—H	H···*A*	*D*···*A*	*D*—H···*A*
C4—H4A···Cl1^i^	0.93	2.81	3.663 (6)	153
C5—H5A···Cl1^ii^	0.93	2.83	3.558 (7)	136
C10—H10A···F1^iii^	0.93	2.41	3.046 (8)	126

Symmetry codes: (i) *x*+1/2, −*y*+1/2, −*z*+1; (ii) −*x*−1/2, −*y*, *z*+1/2; (iii) −*x*+1/2, −*y*+1, *z*−1/2.
